# P2X7 Receptor as a Key Player in Oxidative Stress-Driven Cell Fate in Nonalcoholic Steatohepatitis

**DOI:** 10.1155/2015/172493

**Published:** 2015-03-01

**Authors:** Saurabh Chatterjee, Suvarthi Das

**Affiliations:** Environmental Health and Disease Laboratory, Department of Environmental Health Sciences, University of South Carolina, Columbia, SC 29208, USA

## Abstract

Incidences of nonalcoholic fatty liver disease parallels increase in the global obesity epidemic. NAFLD has been shown to be associated with risks of cardiometabolic disorders and kidney disturbances. It is accompanied by insulin and leptin resistance that complicate the diagnosis and treatment of this public health menace. Though significant research is underway for understanding the molecular mechanisms of NAFLD and its subsequent inflammatory and fibrotic manifestations like nonalcoholic steatohepatitis, the role of purinergic receptors has been unclear. It is increasingly being recognized that damage associated molecular patterns like NAD and ATP that are released from injured cells via hepatocellular injury either by oxidative stress or lipotoxicity from steatosis activate the purinergic receptor. Based on evidence from inflammatory responses in the airways and vasculature and autoimmune complications in humans and rodents, it is beyond doubt that hepatocellular inflammation such as that seen in NASH can result from the activation of purinergic receptors. This event can result in the formation of inflammasomes and can be an important pathway for the progression of NASH. The present review evaluates the current knowledge of the role of oxidative stress and its signaling via P2X7 receptors in hepatocellular injury that might contribute to the NASH pathophysiology.

## 1. Introduction

The “Global Society” is driving us towards a global epidemic of obesity, type 2 diabetes mellitus (T2DM), and metabolic syndrome (MS), with each passing day. Insulin resistance is the most closely associated pathophysiological hallmark [1–3], whereas nonalcoholic fatty liver disease (NAFLD) is the hepatic manifestation of the metabolic syndrome [4]. The estimated worldwide prevalence of NAFLD is 6.3%–33% with a median of 20% in the general population. However, in the presence of obesity and T2DM, the prevalence of NAFLD increases to about 75% [5–8]. NAFLD will be the most important chronic liver disease within a few years posing a grave challenge for the gastroenterologists and the hepatologists worldwide [9, 10]. The complex spectrum of NAFLD constitutes of benign steatosis, more severe alterations like NASH (nonalcoholic steatohepatitis), cirrhosis, and sometimes hepatocellular carcinoma [10, 11]. The pathogenesis proceeds from accumulation of fat in the liver followed by liver injury, inflammation, and fibrosis and then scarring of liver, till the scar tissue replaces the liver cells, giving rise to the cirrhotic phase, primarily resulting from an aberrant tissue repair process [11]. Cirrhosis is irreversible and if untreated; the cirrhotic liver can progress to hepatocellular carcinoma. The mechanisms that account for disease progression in NAFLD are still poorly understood. Yet a crucial stage that might be treated as an early indicator for the initiation of NASH from benign steatotic and usually asymptomatic liver might be that of sinusoidal endothelial injury [12].

## 2. The Concept of 2nd Hit and Multiple Hits: Roles of Oxidative Stress and Proinflammatory Signaling Pathways

The specific stages or the mechanistic pathway for the progression of fatty liver to NASH is based on theories till date. The “two-hit” hypothesis put forward by Day and James in 1998, recognizes lipid accumulation in the hepatocytes as the first hit, characterized by unaltered lipid metabolism in the liver [13]. The hypothesis evolved from the study which showed that ob/ob mice and fa/fa rats were exquisitely vulnerable to LPS-induced liver injury and concluded further that fatty livers were susceptible to liver injury from secondary stressors [14]. Later study from Yamaguchi et al. showed that hepatocyte triglyceride accumulation per se was not detrimental but inhibition of triglyceride synthesis exacerbated liver injury [15]. Rather, triglyceride accumulation was a protective mechanism that buffered hepatocytes from toxicity of nonesterified fatty acids that flooded the liver following insulin resistance [16]. Obesity-induced insulin resistance which likely arises from the effects of TNF-*α* has been observed to be a key pathogenic factor for the development of hepatic steatosis [1, 17–19]. One would argue that hepatic steatosis might be contributing to the underlying condition of hepatocyte stress based on evidence that polymorphisms of PNPLA3 are a major risk factor for NASH and cirrhosis. The 2nd of the two hits could be due to (i) oxidative stress, (ii) proinflammatory cytokines and adipokines, (iii) mitochondrial dysfunction, or (iv) endoplasmic reticulum stress. Any one of these 2nd hits could lead to hepatocyte injury, inflammation, and then fibrosis. In 2010, a more holistic model of “multiple parallel hits” was proposed by Tilg and Moschen wherein many parallel hits derived from the gut and/or the adipose tissue that may promote liver inflammation were identified [20]. Endoplasmic reticulum stress and related signaling networks, (adipo)cytokines, and innate immunity are emerging as central pathways that regulate key features of NASH.

## 3. Oxidative Stress in NASH Progression

Progression from NAFLD to NASH is multifactorial and there have been implications of oxidative stress as a crucial trigger for NASH, although a more specific cause-effect relationship between oxidative stress and NASH has not yet been established [21–23]. Increased levels of reactive oxygen species and lipid oxidation products and decreased levels of antioxidant enzymes such as superoxide dismutase (SOD) and catalase and antioxidant compounds such as glutathione have been observed in patients of NAFLD/NASH compared with those observed in the healthy subjects. Various kinds of oxidants have been reported to cause lipid oxidation: enzymatic oxidants namely cytochrome P450, lipooxygenase, cyclooxygenase and so forth, and/or nonenzymatic oxidants such as free radicals, hypochlorite, or singlet oxygen might have singular or synergistic effect in causing oxidative stress [21, 24]. CYP2E1, an isoform of cytochrome P450, expressed in the liver, has emerged as an important cause of ROS overproduction, as shown in both rodent and human models [25, 26]. Lipid oxidation products render damage in multiple ways: they are cytotoxic, cause damage to proteins and DNA, and often act as ligands or antagonists for various receptors and thus modulate inflammation [21, 27, 28]. The levels of oxidative stress markers such as 4-hydroxynonenal and 4-hydroxydeoxyguanosine correlate well with the severity of necroinflammation and subsequent fibrosis [29–31]. In this scenario, recent evidence indicates that lipid peroxidation products originating from the oxidation of phospholipids can act as damage associated molecular patterns (DAMPs) and promote inflammation through the interaction with soluble and cell-associated pattern recognition receptors [32, 33]. Mitochondria are the most important cellular source of ROS, and mitochondrial dysfunction might therefore be a common denominator in the pathological mechanisms of NASH [34]. Although the mechanisms of mitochondrial dysfunction are not clearly understood, emerging data suggest that ROS, lipid peroxidation products are involved in the second hit namely oxidative stress, which induces the progression of simple steatosis to NASH. Furthermore, ROS induce the directional migration of resident hepatic profibrogenic cells, resulting in liver fibrosis [35–37]. Considering oxidative stress as a second hit might be a feasible starting point, but it would be reasonable to assume that multiple, rather than single, prooxidative intracellular and extracellular triggers act in conjunction promoting oxidative stress that drives the development of NASH [34]. Recent reports have shown the involvement of peroxynitrite as an important reactive species in NASH pathogenesis. Chatterjee et al. have shown that peroxynitrite from NADPH oxidase activates Kupffer cells and causes inflammation in the steatotic liver [38]. Unpublished reports from our laboratory have shown the involvement of peroxynitrite in causing toll like receptor 4 trafficking into the lipid rafts, a significant event in TLR4 activation and signaling in NASH. The trafficking process was attenuated by peroxynitrite scavenger phenyl boronic acid (FBA) that has been reported to be specifically reacting with peroxynitrite [39]. Though the involvement of NADPH oxidases have been shown in NASH pathophysiology, especially in fibrosis related complications, the molecular basis of NADPH oxidase mediated ROS generation is now beginning to evolve following the use of species-specific probes like FBA and a better understanding of the free radical chemistry that might trigger the generation of reactive oxygen and nitrogen species in NASH. However, it remains to be seen whether the sequence of events following hepatocyte necrosis via lipotoxicity is causing innate immune responses and subsequent oxidative stress or vice versa.

## 4. Oxidative Stress and Hepatocyte Degeneration/Necrosis

ROS generated by CYP2E1 and other sources might induce damage in two ways: first, as stated before by direct modulation of cellular functions by modification/peroxidation of lipids, proteins, and DNA [40, 41]. The second mechanism could be the activation of the cell-death signaling pathways. This activation might either be from ROS or reactive byproducts of lipid peroxidation, 4-HNE in particular, which has been shown to activate JNK [41–44]. Lipotoxicity is now a well-accepted trigger for metabolic syndrome related NASH [45–48]. In contrast to the levels of saturated free fatty acids (which might induce hepatocyte degeneration via JNK-mediated mitochondrial injury), that often do not differ between NASH and “non-NASH” phenotypes, the levels of biologically reactive free cholesterol (FC) are almost always high in NASH compared to benign steatosis [49–52]. In their report, Gan et al. suggest that FC causes hepatocyte apoptotic and necrotic cell death that depends on JNK activation, mitochondrial injury with mitochondrial permeability transition (MPT), ATP depletion, oxidative stress, and caspase-3 activation, but not on ER stress [52]. It is also implicated that JNK1 mediates apoptosis and necrosis and that HMGB1, an archetypical danger-associated molecular pattern (DAMP) molecule, contributes to hepatocellular injury that may be amplified and perpetuated via TLR4 and JNK1 [52]. In another report by Amir et al., JNK 2 has been observed to be hepatoprotective whereas the downstream effector molecule c-Jun causes hepatocyte death by manipulating the cell signaling pathway [53]. In summary, it can be credibly suggested that although the molecular mediators for the pathway are still to be elucidated, oxidative stress is a potent initiator of hepatocyte degeneration or necrosis resulting in the release of DAMPS or alarmins in the microenvironment surrounding the affected cells. Apoptosis has long been regarded as a noninflammatory or even anti-inflammatory mode of cell death, but recent studies suggest that this is not always the case. Necroptosis is a programmed form of necrosis that is engaged under certain conditions when caspase activation is blocked. Necroptosis is also regarded as a highly proinflammatory mode of cell death [54].

## 5. Release of ATP: ATP and NAD as DAMPs

Endogenous molecules and fragments from damaged cells and tissues can be recognized as danger signals, referred to as damage-associated molecular patterns (DAMPs). DAMPs are used for damage-self recognition to evoke immune inflammatory responses and damage healing independent of but in cooperation with exogenous danger signals [55]. In events such as nonspecific cytolysis of healthy cells in physical or stress-related trauma, endogenous molecules such as ATP (3–5 mmoles) may be released extracellularly and can serve as damage-associated molecular patterns (DAMPs) [56]. Increased release of DAMPs can result in cytolysis or necrotic cell death, which generates profound sterile inflammation characterized by accumulation of neutrophils and other immune effector cells [57]. The endogenous or self-molecules (extracellular matrix proteins (ECM), calcium-binding proteins, and structural proteins) typically function in normal cell homeostasis but are also recognized as danger signals when released into the extracellular space [58, 59] exposing hydrophobic portions of the molecules that are normally hidden in healthy living cells [55]. Matzinger and others have extended the danger model as more has been learned about the role of danger signals in tissue injury and other diseases [55, 60–62].

## 6. P2X7r Stimulation

P2X7r (Purinergic receptor X7) is an atypical member of the purinergic receptor family which when receives a stimulus from a ligand like ATP, an intrinsic ion channel opens up followed by recruitment of a hemichannel protein called pannexin-1. The macrophages which are deficient in P2X7r have been shown to be unresponsive to ATP as a ligand, corroborating the role of P2X7r in transmission of the stimulus from such extracellular ligands. It is widely accepted that cytosolic potassium is the key signaling molecule that initiates caspase-1 activation via K efflux from the cell. Evidence for this pathway is largely based on the ability of elevated extracellular K to prevent NALP3 inflammasome activation or enzyme activity assays in low K salines [63–65]. Recent data suggest that a second pathway, involving NADPH oxidase, may contribute to caspase-1 activation.

It has also been observed that there is an extracellular ATP-triggered, TNF-*α* release in microglia, the resident macrophages in the brain, mediated by the P2X7 receptors [66]. Recent reports indicate that the ATP-triggered P2X7 receptor might target NADPH oxidase via extracellular calcium influx, p38 MAPK and PI3 kinase activity, but the sites of these events have only been observed in microglia-induced cortico-neuron injury [67]. The augmentation of P2X7 receptor-induced NADPH oxidase activity has been shown in endotoxin-primed human monocytes [63]. The same study also reported the formation of peroxynitrite from nitric oxide and superoxide released from NADPH oxidase following ATP stimulation in these cells [63, 68].

## 7. P2X7r in Innate Immunity

Induction of P2X7r by ATP can lead to the maturation, chemotaxis, and finally release of TH17 biasing cytokines, IL1*β*, and IL6. Thus, P2X7r has been shown to play a crucial role in the initiation of innate proinflammatory inflammation, DC17 differentiation and Th17-biased immunity [69]. P2X7r's role in a potential modulator of angiogenesis or wound repair has also been reported [70]. Thus, based on the proof available, it would not be wrong to conclude that P2X7r acts as an innate immune modulator in NASH progression.

## 8. P2X7r in Cell Fate in NASH

Cellular injury plays a central role in triggering inflammation in NAFLD and facilitates its progression to the inflammatory phenotype as seen in NASH and the later fibrotic pathology [71–73]. Normal healthy liver lobules are with little or no accumulation of fat in hepatocytes. On the other hand, stellate cells are laden with fat and start to lose fat when they are in activated state. Normal livers hardly possess activated Kupffer cells (the resident macrophages). NAFLD livers and livers from alcoholic liver injury, CCl4 poisoning, toxin exposure and drug-induced hepatotoxicity show distinct fat accumulation in hepatocytes are largely seen as vacuoles within the cells. Steatosis can be both present in macro- or microvesicular forms. Microsteatosis is likely an epiphenomenon that may be a consequence of acute mitochondrial dysfunction and may not be a direct result of hepatocyte injury. The macrovesicular fat accumulation may or may not be causing lipotoxicity and studies show that triglyceride accumulation can be hepatoprotective [16]. The increased toxicity of the accumulating lipids caused by either the failure of triglyceride synthesis, transport or storage result in intrinsic cellular signaling that end in deciding the cellular fate. Many studies report the presence of apoptosis, caspase-independent apoptosis, autophagy, mitophagy, and necrosis. The role of P2X7r has been implicated in the Kupffer cell activation and inflammation, following the release of ATP from necrosed cells in a CCl_4_-induced model of NASH [74]. According to reports, P2X7r acts as a key regulator in modulating oxidative stress-induced autophagy process [75]. The Das et al. study reports that, when P2X7r is stimulated by ATP, then it modulates autophagy by depleting LC3B which is an early autophagy marker [75]. At the same time the protein levels of HSC70 and lysosomal membrane association of LAMP2A were marginally upregulated pointing towards chaperone mediated autophagy and release of autophagolysosomes in the extracellular matrix, thus leading to inflammation [76]. Metabolic oxidative stress also, mainly characterized by oxidatively modified proteins, has been known to induce chaperone-mediated autophagy, increased substrate translocation by HSC 70 toward the lysosomal membrane, and increased LAMP2A levels [77]. There exists a correlation between increased lysosomal association of LAMP2A, increase in inflammation and NASH pathophysiology [75, 78, 79]. Das et al. did not report LAMP2A depletion, whereas there was an earlier report by Fortunato and Kroemer emphasizing the depletion of LAMP2A that also stated that impaired lysosomal membrane association and relative paucity of autolysosomes reduced ATP levels and finally resulted in a shift from apoptosis to necrosis [80]. Since defective autophagy results in depletion of LC3B, and a decrease in LC3B levels has been already proven to be a driver of caspase 1 activation, thus indicating a shift towards apoptosis and inflammation, it can be well assumed that P2X7r acts as molecular switch crucial for deciding the cell fate also the programmed pathway of cell survival/death in NASH, before it finally moves on to necrotic damage [81].

## 9. Conclusions

**Figure 1:**
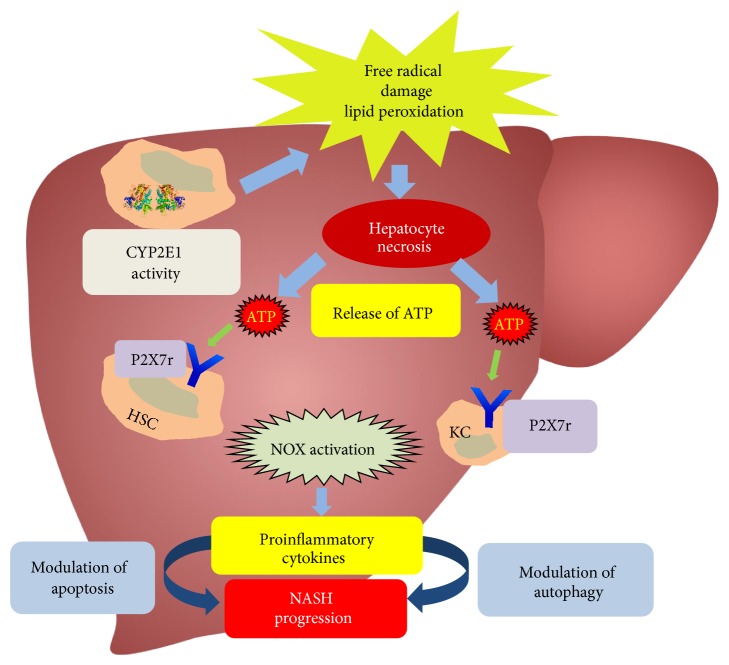
Mechanism of CYP2E1-induced oxidative stress and altered cell fate in NASH.

As an ion channel protein that is responsive to ATP and NAD at the cellular membrane, P2X7 receptor might play a key role in modulating the cell fate in NASH. As described in the review, oxidative stress either in the hepatocytes themselves due to lipotoxicity or metabolic disturbances because abnormal fat storage can trigger the release of damage associated molecular patterns into the highly mobile extracellular space that can activate nearby resident macrophages, endothelial cells, or stellate cells. Based on the recent findings that P2X7 receptors are located on the membranes of all the cell types of the liver sinusoidal area, it may be assumed that the ion channel protein will be activated on these cells [75]. Further, these receptors can trigger the activation of NADPH oxidase isoforms, especially NOX2 and NOX4 in the sinusoidal cells to cause their activation and result in uninterrupted innate immune signaling, release of proinflammatory cytokines. Since the proinflammatory events have been correlated well with the cellular longevity and fate in the liver microenvironment, it is justifiable to assume that depending on the nature of insult the liver cells will undergo apoptosis, autophagy, or necrosis. The chain of events that P2X7 receptor stimulation may trigger and a detailed understanding of its pathway that covers oxidative stress-innate immune crosstalk may serve as a crucial benchmark in understanding NASH pathophysiology and help design new therapeutic strategies for this metabolic disease (Figure 1).
